# The psychometric properties of the Bidimensional Acculturation Scale for Marriage-Based Immigrant Women in Taiwan

**DOI:** 10.1371/journal.pone.0258323

**Published:** 2021-10-12

**Authors:** Hung-Hui Chen, Jerry Cheng-Yen Lai, Li-Yin Chien

**Affiliations:** 1 School of Nursing, College of Medicine, National Taiwan University, Taipei, Taiwan; 2 Department of Nursing, National Taiwan University Hospital, Taipei, Taiwan; 3 Department of Medical Research, Taitung MacKay Memorial Hospital, Taitung, Taiwan; 4 Department of Artificial Intelligence and Medical Application, MacKay Junior College of Medicine, Nursing, and Management, New Taipei City, Taiwan; 5 Institute of Community Health Care, College of Nursing, National Yang Ming Chiao Tung University/Yang-Ming Campus, Taipei, Taiwan; University of Bologna, ITALY

## Abstract

**Introduction:**

Marriage-based immigrant women are increasing around the world. Although bi-dimensional acculturation is important for immigrant women’s health, the existing scales have mainly been developed for immigrant women in Western countries and hence some items may not be suitable for Asian contexts. Thus, we developed and evaluated the Bidimensional Acculturation Scale for Marriage-Based Immigrant Women (BAS-MBIW) in Taiwan.

**Methods:**

The BAS-MBIW was developed based on a literature review and clinical observations. Bi-dimensional acculturation involves “adaptation to host culture (acculturation)” and “maintenance of heritage culture (enculturation).” The initial scale included two 24-item subscales. The validation samples were 310 marriage-based immigrant women who were pregnant for at least twelve weeks in Taiwan. The BAS-MBIW was assessed and modified by experts. Data analyses included factor analysis, Pearson’s correlation, and Cronbach’s alpha coefficient.

**Results:**

Expert reviews and factor analysis indicated that the scale had acceptable content and construct validity. The validated scale includes two 19-item subscales, encompassing six domains: language, media, food preference, cultural heritage, social interaction, and shopping and merchandise preference, with good internal consistencies (Cronbach’s alpha coefficient is 0.88 for acculturation and 0.83 for enculturation). Acculturation was positively related to local language ability and duration of immigration but negatively related to age at immigration, stress, and depression; whereas enculturation was positively related to age at immigration, stress, and depression but negatively related to duration of immigration, indicating convergent validity.

**Conclusions:**

The BAS-MBIW offers reliable and valid assessments of pregnant immigrant women’s level of acculturation and enculturation in Taiwan. The BAS-MBIW could be used to assess bi-dimensional acculturation among marriage-based immigrant women.

## Introduction

In the past few decades, marriage-based immigration has increased in countries throughout the world [1, 2]. The numbers of marriage-based immigrants have grown rapidly in Japan, Taiwan, and South Korea since the1980s. This may be due to the high economic development and government policies of these countries [3]; most marriage-based immigrants to these countries are women from Southeast and East Asia [3]. More than 500,000 foreign women have married Taiwanese men and immigrated to Taiwan by December 2019 [4]. In Taiwan, most marriage-based immigrant women come from China (67%), Vietnam (21%), or other Southeast Asian countries, including Indonesia, the Philippines, Thailand, and Cambodia (11%) [4].

Many marriages with Southeast Asian immigrant women in Taiwan are arranged through friends, relatives, or international marriage agencies. Researchers believe that these marriages generally do not treat love and affection as the foundation of the relationship [5, 6]. Women in these relationships usually immigrate to a foreign land (Taiwan) by themselves, without their original family and friends [7]. More than 90% of marriage-based immigrant women give birth to a child within the first or second year of their marriage [8, 9]. These women usually bear the duties of childbirth and play multiple roles (such as wife, daughter-in-law, family caregiver, and mother) shortly after their arrival [10]. In a patrilineal society, immigrant women may not only find themselves in a patriarchal marriage, but also have lower domestic decision-making power and social support than native women [11]. These women are usually expected to assimilate promptly into their husband’s extended family and undertake housekeeping and childbearing duties. They must take care of the entire family, including cooking meal, shopping for daily necessities, and preparing for festival celebration and religious ceremonies. Pregnant immigrant women encounter many Chinese cultural practices that contradict their native cultural practices and beliefs. This can include avoiding scissors and spicy food, sleeping in separate beds, and consuming Chinese herbal soups; the goal is to keep mothers and fetuses physically healthy in Chinese society [5]. Taiwanese government offers local language courses, translation services by request, and welfare services for the immigrants to help immigrants adapt to the host society. However, much less support is extended by the government to help immigrants to maintain their heritage culture.

Immigrants usually face challenges of acculturation, a process of adopting behaviors, attitudes, values and beliefs of a new culture, in a new host country [12, 13]. The process of adapting to a new culture in a host society is known as uni-dimensional acculturation [14–16]. More recently, researchers have employed a more dynamic bi-dimensional view in immigration-related studies, which expands the original uni-dimensional view to accommodate one’s adaption to a host culture (acculturation) as well as one’s maintenance of an original culture (enculturation) [17–19]. Compared to the uni-dimensional version, bi-dimensional acculturation emphasizes cultural diversity in a multicultural society [20, 21]. Bi-dimensional acculturation depicts each ethnicity as a component of a mosaic puzzle or a salad platter in a pluralistic society. The bi-dimensional acculturation model treats adaptation to host culture and maintenance of heritage culture as two independent dimensions [12, 13, 19, 22].

Traditional immigration-related variables such as duration of immigration, local language ability, and age of immigration were well correlated to one’s level of adaptation to host culture in previous studies [7, 23–25], therefore, they were correlated to the newly developed scale in this study. However, the association between level of enculturation and immigration-related variables has not been clarified in previous studies. Nonetheless, older age at immigration means longer exposure to heritage culture, whereas longer duration of immigration may impose difficulty in maintaining the heritage culture since marriage-based immigrant women marry into families of local culture. Therefore, we hypothesized that older age at immigration and shorter duration of immigration will be correlated with higher level of maintenance of heritage culture. Higher level of adaptation to host culture has been related to better psychological wellbeing among immigrants [7, 26]; whereas higher level of maintenance of heritage culture may be associated with worse psychological wellbeing in countries with strict rules regarding citizenship such as Taiwan [27].

Several bi-dimensional acculturation scales have been developed and used in immigration studies [28–32]. However, the existing bi-dimensional acculturation scales were mainly developed for immigrants in Western countries [28–32]. Although researchers have translated the scales originally developed for Southeast Asian immigrants in Western countries and validated them in Asian countries, they have had to exclude several items to maintain good psychometric properties while applying the scale to local Asian contexts [33]. As a result, the existing instruments often have limited domains and operational items, and do not assess a range of acculturation behaviors and situations.

For example, food culture and Chinese medicines are well-known in Taiwanese culture and could be used as indicators to reflect the level of acculturation. In addition, marriage-based immigrant women have been reported to prefer using daily necessities or cosmetics of specific brands, from specific places of production, or sold in specific stores. Shopping and merchandise preference could thus be applied to reflect the level of bi-dimensional acculturation. There is therefore an impetus to design an instrument of bi-dimensional acculturation for marriage-based immigrant women in Taiwan.

We focused on pregnant women, because pregnancy is an important starting point for an immigrant woman to develop a strong connection with her husband’s family and Taiwanese society as it is a culturally sensitive period in most Asian cultures. The study objectives were to develop and evaluate a new scale to measure bi-dimensional acculturation among marriage-based immigrant women and assess its psychometric properties among marriage-based pregnant immigrants in Taiwan.

## Methods

### Design

This study was part of a three-year study aimed at examining depression trajectories from pregnancy to one year postpartum among native and immigrant women in Taiwan. This study was a cross-sectional questionnaire survey undertaken from March 2013 through September 2014 to validate the instrument prior to the main cohort study. Data were collected through face-to-face and telephone interviews based on each study participant’s convenience. The research assistants received three hours of training in data collection, interviewing skills, administering the questionnaire alone or with a trained translator, and answering questions. We used interviews rather than self-administered surveys since listening comprehension is usually much better than reading ability among immigrant women. Immigrant women were assessed on their ability to understand Mandarin Chinese in the interviews. If the language ability of immigrant women was limited, a trained interpreter would assist the interview. The study protocol was approved by the institutional review boards at Mackay Memorial Hospital, Tzu Chi General Hospital Taipei Branch, Taipei City Hospital, and Saint Mary’s Hospital Luo-Dong in Taiwan.

### Study participants

The inclusion criteria for this study were pregnant immigrants who were born outside of Taiwan and married to Taiwanese men, at least 20 years of age, pregnant for more than 12 weeks, and currently living in Taiwan. Those immigrant women who had serious perinatal complication or risk of miscarriage were excluded. The research team cooperated with Health Centers and obstetric clinics to recruit study participants. The staff at the collaborating sites referred potential study participants to our team. Subsequently, a research assistant approached the eligible women to explain the study, sign a consent form, and arrange for interviews. A total of 372 eligible women were referred to the research team, but 62 women refused to participate in this study. Finally, 310 pregnant immigrant women were included in this study.

### Measurement

Based on a literature review and our observations, we developed the Bidimensional Acculturation Scale for Marriage-Based Immigrant Women (BAS-MBIW) to reflect the attitudes and behaviors of immigrant women toward the necessities of everyday life. The BAS-MBIW has two separate, parallel 24-item subscales to measure an individual’s adaptation to a host culture (acculturation) and maintenance of heritage culture (enculturation). Each item is rated on a 5-point Likert scale (0–4). A higher total score on one of the subscales indicates a higher level of acculturation or enculturation. These 24 items reflected the following seven domains: language use, media use, food preference and use, cultural heritage, social interaction, national identity, and shopping and merchandise preference [34–37]. Language use refers to the language used in communication and thinking. Media use refers to audiovisual and print media use, and singing in dialects. Food preference and use refers to the preference for and eating food/cuisine. Cultural heritage refers to acceptance of traditional customs, festivals, folk beliefs and traditional medicines. Social interaction refers to interaction with friends from host or heritage society. National identity refers to self and others’ identification of the immigrant’s nationality. Shopping and merchandise preference refers to the preference for daily necessities or cosmetics of a specific brand, from a specific place of production, or from a specific store.

The items of the BAS-MBIW are listed in Table 1. Besides the self-developed items, several items were adapted from the concept of other scales. Items 1, 2, and 3 were adapted from Anderson et al. [38]. Items 4 and 5 were adapted from Barona and Miller [39]. Items 5 and 17 were adapted from Briman and Tyler [40]. Items 7 and 9 were adapted from Nguyen and von Eye [41]. Items 10 and 11 were adapted from Tsai, Ying, and Lee [32]. Items 13 and 14 were adapted from Gim Chung et al. [42]. Item 15 was adapted from Klonoff and Landrine [43]. Item 18 was adapted from Chen et al. [5]. Items 21–24 were inspired from Gim Chung et al. and Mavreas, Bebbington, and Der (1989) [42, 44].

**Table 1:** Exploratory factor analysis of the acculturation and enculturation subscales in the Bi-dimensional Acculturation Scale for Marriage-Based Immigrant Women (BAS-MBIW).

	Item	Acculturation subscale			Enculturation subscale		
		Factor loading	Eigenvalue	% of variance	Factor loading	Eigenvalue	% of variance
Language use			2.6	12.77		5.2	26.12
1.	How often do you speak Mandarin or Taiwanese/your native language with adult family members?	0.95			0.96		
2.	How often do you speak Mandarin or Taiwanese/your native language with young family members?	0.93			0.96		
3.	How often do you speak Mandarin or Taiwanese/your native language with neighbors, friends, or co-workers?	0.94			0.95		
4.	How often do you use Mandarin or Taiwanese/your native language to think/memorize?	0.84			0.93		
Media use			2.0	9.95		2.5	12.74
5.	How often do you watch local/your mother country’s TV channels?	0.62			0.86		
6.	How often do you listen to local/your mother country’s radio stations or music?	0.88			0.87		
7.	How often do you read local/your mother country’s newspapers, magazines or books?	0.88			0.87		
8.	How often do you sing in local/your mother country’s dialects?	0.88			0.81		
Food preference and use			1.8	8.96		1.4	7.18
9.	How often do you enjoy local/your mother country’s cuisine?	0.86			0.72		
10.	How often do you choose local/your mother country’s cuisine when you dine out?	0.87			0.87		
11.	How often do you eat local/your mother country’s food at home?	0.89			0.86		
12.	How often do you consume local/your mother country’s supplements or tonic soups? ^b^	-			-		
Cultural heritage			1.7	8.29		1.9	9.25
13.	How often do you follow Taiwanese/your mother country’s traditional rituals?	0.80			0.92		
14.	How often do you enjoy Taiwanese/your mother country’s festivals?	0.88			0.94		
15.	How often do you accept Taiwanese/your mother country’s folk beliefs?	0.89			0.92		
16.	How often do you use Taiwanese/your mother country’s traditional medicine when you’re sick? ^b^	-			-		
Social interaction			1.3	6.66		1.3	6.63
17.	How often do you interact with friends who are native Taiwanese/from your mother country?	0.97			0.97		
18.	How often do you ask friends who are native Taiwanese/from your mother country for opinions or assistance?	0.97			0.97		
National identity			-	-		-	-
19.	How often do you think that you are part of the Taiwanese/people of your mother country?^a^	-			-		
20.	How often do friends from your mother country think that you are part of the Taiwanese/people of your mother country?^a^	-			-		
Shopping and merchandise preference			6.9	34.35		4.0	20.16
21.	Do you prefer shopping in a store owned by a person who is Taiwanese/from your mother country?	0.95			0.89		
22.	How often do you shop in a store owned by a person who is Taiwanese/from your mother country?	0.97			0.89		
23.	Do you prefer using manufactured merchandise that are Taiwanese/from your mother country?	0.95			0.90		
24.	How often do you choose to purchase manufactured merchandise that are Taiwanese/from your mother country?	0.90			0.90		
Cumulative % of variance				80.98			82.07

*Note*. The underlined section refers to the part that differs between the acculturation and enculturation subscales. For the acculturation subscale, the word or phrase before the **/** is the item. For the enculturation subscale, the word or phrase that follows the / is the item.

Each item was rated on a 5-point Likert scale from 0–4 for rarely, seldom, sometimes, often, and usually, respectively.

^a^Item 19 and 20 were deleted because the item-total correlations were 0.22 (less than 0.3) for the enculturation subscale.

^b^Item 12 and 16 were loaded in a different factor for the enculturation subscale, and thus were deleted from further analysis.

Sociodemographic variables included age (years), birth country, educational status, work status, and family income. Traditional indicators for acculturation, such as age at immigration, local language ability, and duration of living in Taiwan, were also included.

Stress was measured by the 10-item Perceived Stress Scale (PSS) with a Likert scale ranging from 0 (none) to 4 (high) for each item [45, 46]. Higher scores indicate greater levels of perceived stress.

Depression was assessed using the Edinburgh Postnatal Depression Scale (EPDS) [47], which is a 10-item instrument measuring depressive symptoms in the past seven days [48]. The scores range from 0 to 30, with higher scores indicating higher levels of depressive symptoms. The validity and reliability of the EPDS has been demonstrated among immigrant women in Taiwan [49].

### Data analysis

The content validity of the scale was confirmed through expert judgement, while construct validity was presented by factor analysis. Convergent validity was assessed using Pearson’s correlation coefficient. Cronbach’s alpha was used to assess the internal consistency of the scale. Five experts of different specialization (social science, public health, nursing, and medicine) evaluated the content validity of the scale, and reviewed each item for its completeness, clarity, and consistency. The content validity index (CVI) scoring sheet was used to rate item relevancy using a 4-point Likert scale (ranging from 1: not relevant to 4: highly relevant). Items with a score of less than 3 were revised according to the experts’ suggestions. Item-level CVI (I-CVI) was calculated by dividing the number of experts who rated an item with a score of 3 or 4 with the total number of experts. Scale-level CVI (S-CVI) was computed by dividing the number of items that received a relevance rating of 3 or 4 on a scale by all the experts with the total number of items [50]. An I-CVI and S-CVI of 80% was deemed acceptable [50].

Item-total correlation was used to examine the related association of one item to the total scale [51]. A value of ≥0.3 was considered acceptable [52]. Internal consistency was assessed by Cronbach’s alpha. Cronbach’s alpha ≥ 0.7 was considered to be acceptable [53].

We performed exploratory factor analysis (EFA) on both the subscales to investigate the possible factor structure based on a set of observed variables and to simplify the interrelated measure [54]. Kaiser-Meyer-Olkin (KMO) and Bartlett’s tests were used to assess the data suitability for employing EFA. Factors were extracted using principal components factor analysis with promax rotation and Kaiser’s eigenvalue of greater than 1. In EFA, the percentage of total variance explained in response to the questionnaire should be at least 50% [55]. Only items that belonged to a specific factor with a factor loading of greater than 0.5 in the EFA analysis were retained.

Confirmatory factor analysis (CFA) was performed to examine the hypothesized interrelated measures and factor structure based on the selected set of observed variables from the EFA analysis [54]. CFA was conducted for acculturation and enculturation subscales separately. The goodness of fit for CFA models was assessed using relative chi-square (χ2/df), the comparative fit index (CFI), the Tucker Lewis Index (TLI), and the root mean square error of approximation (RMSEA). A relative chi-square of less than 3 was deemed acceptable in our study. A value ≥0.9 for the CFI and the TLI signaled good model fit. An RMSEA range from 0.08 to 0.10 signaled a mediocre fit and a value ≤0.08 indicated a good fit.

Traditional indicators for acculturation, such as age at immigration, local language ability, and duration of immigration, were correlated with an individual’s level of acculturation [23–25]. In addition, level of bi-dimensional acculturation may be associated with stress and depression among immigrants [56, 57]. Pearson’s correlation coefficient was used to examine convergent validity. Data analyses were performed using the computerized statistics software package IBM SPSS Statistics 21.0 (IBM Corp., Armonk, NY, USA) and LISREL 8.80.

## Results

The I-CVIs of all items ranged from 0.6 to 1.0, while the S-CVI of both the scales was 0.83. Four original items (Items 21–24) with scores of less than 3 were revised after discussing and confirming with the experts. The face validity of the BAS-MBIW was determined by face-to-face interviews with ten marriage-based immigrant women who had lived in Taiwan for more than five years. We used their perspectives as immigrant women to revise the item descriptions and improve the readability and clarity of certain sentences.

The characteristics of the 310 participants are presented in Table 2. Most of the women were 25–35 years of age (73.5%). About one-fourth (24.6%) had an educational level of junior high school or less. More than 70% were unemployed. About 14% perceived their family income as insufficient. The study immigrants were mostly from China (68.4%), followed by Vietnam (16.1%). More than half (57.8%) had immigrated to Taiwan for <3 years ago.

**Table 2:** Characteristics of the study participants (N = 310).

Variables	n	%
Age		
<25 y	39	12.6
≥25 to <30 y	129	41.6
≥30 to <35 y	99	31.9
≥35 to <40 y	37	12.0
≥40 y	6	1.9
Educational level^†^		
Elementary school or lower	13	4.2
Junior high school	63	20.4
Senior high school	128	41.4
University or higher	105	34.0
Work status		
None	220	71.0
Part-time	22	7.1
Full-time	68	21.9
Family income		
Very insufficient	3	1.0
Insufficient	41	13.2
Just making a living	141	45.5
Sufficient	124	40.0
Very sufficient	1	0.3
Birth country		
China	212	68.4
Vietnam	50	16.1
Myanmar	14	4.5
Indonesia	13	4.2
Malaysia	7	2.3
Philippines	4	1.3
Thailand	2	0.6
Cambodia	2	0.6
Nepal	1	0.3
Japan	3	1.0
Canada	1	0.3
Poland	1	0.3
Age at immigration^‡^		
<25 y	125	40.6
≥25 to <30 y	117	38.0
≥30 to <35 y	57	18.5
≥35 to <40 y	9	2.9
Duration of living in Taiwan^‡^		
<3 y	178	57.8
3 to <5 y	50	16.2
5 to <10 y	45	14.6
10 to <15 y	30	9.7
≥15 y	5	1.6

*Note*.

^†^N = 309.

^‡^N = 308.

All items correlated well with the acculturation subscale, with correlation coefficients ranging from 0.41 to 0.69. For the enculturation subscale, item-total correlations ranged from 0.37 to 0.61, except for items 19 (How often do you think that you are part of the people of your mother country) and 20 (How often do friends from your mother country think that you are part of the people of your mother country). The item-total correlation for both items was 0.22. The two items were deleted from both the acculturation and enculturation subscales to keep the scales parallel.

For the remaining 22 items, EFA was performed. For the acculturation subscale, the chi-squared value of the Bartlett test of sphericity was 5223.50 (df = 231; *p*<0.001), and the KMO coefficient was 0.84. For the enculturation subscale, the chi-squared value of the Bartlett test of sphericity was 5356.46 (df = 231; *p*<0.001), and the KMO coefficient was 0.77. Both indicated that factor analysis could be performed. Items 12 (How often do you consume local/your mother country’s supplements or tonic soups) and 16 (How often do you use Taiwanese/your mother country’s traditional medicines when you’re sick) were removed because factor loading of item 16 was <0.5 for the acculturation subscale; items 12 and 16 did not load on the expected factor for the enculturation subscale. The remaining 20 symmetrical items were retained in the two subscales (Table 1). All factor loadings were greater than 0.5, and all eigenvalues were greater than 1. The six factors explained 80.98% and 82.07% of the total variance for the acculturation and enculturation subscale, respectively.

**Fig 1:**
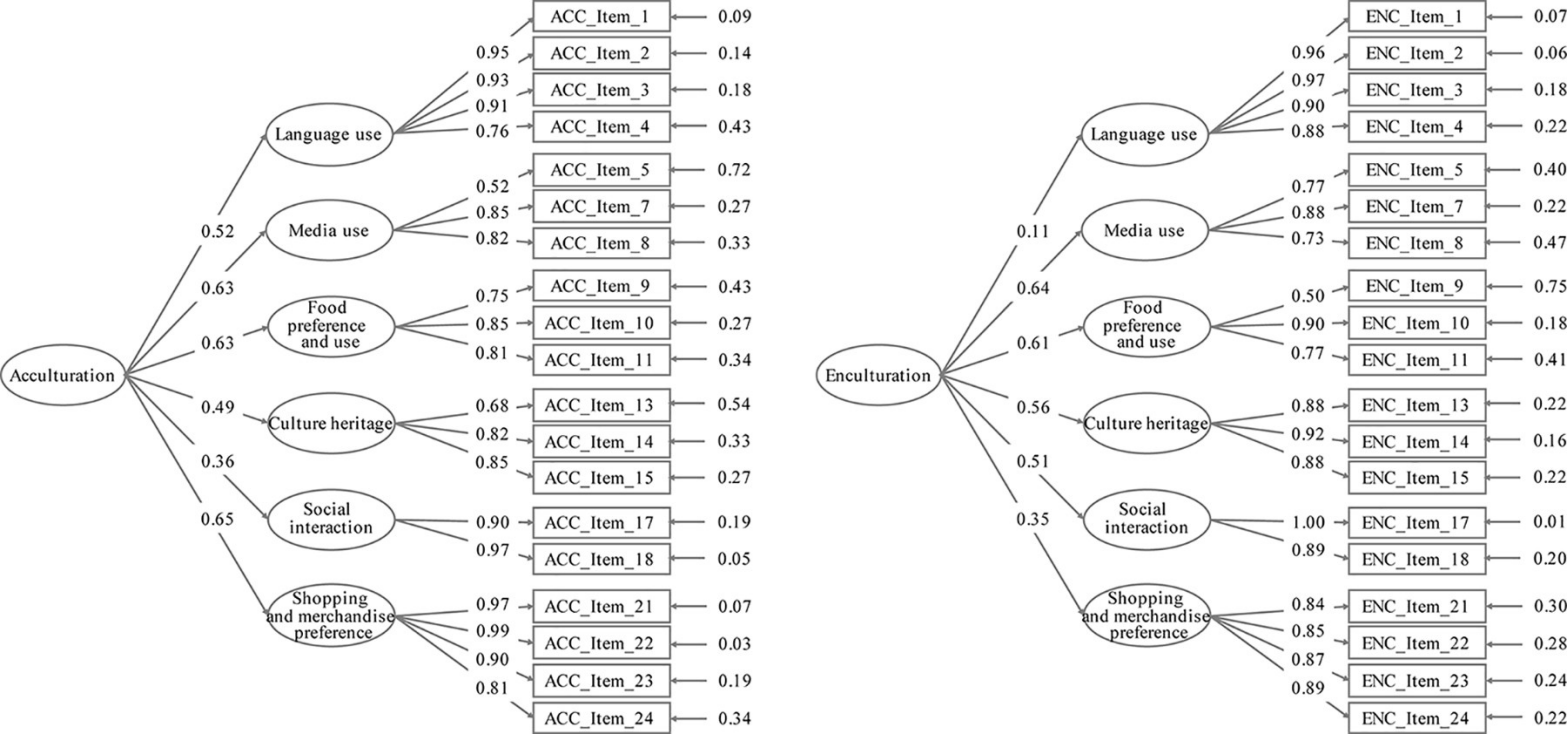
Confirmatory factor analysis for the acculturation and enculturation subscales: Standardized factor loadings and error variances for the second-order six-factor model (N = 310).

The 20-item, second-order and six-factor structure was further examined using the CFA. Item 6 (How often do you listen to local/your mother country’s radio stations or music) was deleted to improve the overall model fit. The CFA results using the remaining 19 items are shown in Fig 1. All factor loadings were >0.3, except for the path between language use and the second-order factor of “enculturation” (0.11, *p* < .05). Since this path was statistically significant and the concept was relevant, we decided to keep the domain. The model fit indices of *χ*^2^/*df*, CFIs, TLIs, and RMSEAs were 2.87, 0.96, 0.95, and 0.078 and 3.03, 0.93, 0.92, and 0.081 for acculturation and enculturation subscales, respectively. The model fit was deemed acceptable.

Means, standard deviations, and inter-correlations of the six-domain and total scores for the two subscales of bidimensional acculturation are presented in Table 3. The six domains correlated well with the overall acculturation and enculturation scores. The only insignificant association occurred between language use and social interaction (*r* = 0.08) in the acculturation subscale. There were three insignificant associations: between language use and food preference and use (*r* = −0.004), language use and social interaction (*r* = 0.03), media use and food preference and use (*r* = 0.10) in the enculturation subscale. The significant correlation coefficients between the six domains of the subscale for acculturation ranged from 0.11 to 0.41. In terms of enculturation, the significant correlation coefficients between the six domains ranged from 0.13 to 0.39. The correlation coefficients between the six domains of the acculturation subscale and that of the enculturation subscale were low on average.

**Table 3:** Means and inter-correlations of the six-domain and total scores for acculturation subscale and enculturation subscale.

**Acculturation**	Mean	SD	0	1	2	3	4	5
0. Total score	54.6	11.66						
1. Language use	13.9	3.81	0.67**					
2. Media use	7.2	3.15	0.69**	0.30**				
3. Food preference and use	8.9	2.63	0.63**	0.32**	0.38**			
4. Cultural heritage	8.5	3.08	0.61**	0.31**	0.29**	0.27**		
5. Social interaction	3.3	2.55	0.47**	0.08	0.27**	0.11*	0.18**	
6. Shopping and merchandise preference	12.7	3.13	0.70**	0.34**	0.37**	0.41**	0.26**	0.28**
**Enculturation**	Mean	SD	7	8	9	10	11	12
7. Total score	33.8	12.20						
8. Language use	10.2	6.30	0.56**					
9. Media use	3.1	2.91	0.58**	0.16*				
10. Food preference and use	6.3	2.66	0.52**	-0.004	0.39**			
11. Cultural heritage	5.4	3.82	0.65**	0.15**	0.31**	0.27**		
12. Social interaction	3.7	2.27	0.51**	0.03	0.25**	0.25**	0.30**	
13. Shopping and merchandise preference	5.1	4.14	0.46**	-0.13*	0.10	0.24**	0.21**	0.28**

*Note*. SD, standard deviation

*p<0.05

**p<0.01.

Cronbach’s alpha coefficients of the 19-item subscales for acculturation and enculturation were 0.88 and 0.83, respectively. Cronbach’s alpha coefficients of the six domains of acculturation, “language use, media use, food preference and use, cultural heritage, social interaction, and shopping and merchandise preference,” were 0.94, 0.77, 0.85, 0.82, 0.93, and 0.96, respectively. The coefficients of the six domains of enculturation were 0.96, 0.83, 0.76, 0.92, 0.94, and 0.92.

The acculturation score (mean = 54.6, SD = 11.66) was higher than that of enculturation (mean = 33.8, SD = 12.20). Acculturation and enculturation scores were not significantly correlated (*r* = −0.01, *p* = 0.84). The correlations between the bidimensional acculturation and related variables are presented in Table 4. Higher acculturation scores were associated with a younger age at immigration (*r* = −0.14, *p* = 0.015), longer duration of living in Taiwan (*r* = 0.24, *p*<0.001), and better Chinese language ability (*r* = 0.40, *p*<0.001). A higher score for enculturation was associated with older age at immigration (*r* = 0.15, *p* = 0.007), shorter duration of living in Taiwan (*r* = −0.11, *p* = 0.048), and better Chinese language ability (r = 0.14, *p* = 0.014). In addition, both acculturation and enculturation had statistically significant correlations with stress and depression. Higher acculturation score was associated with lower stress (*r* = −0.25, *p*<0.001) and depression (*r* = −0.23, *p*<0.001), but higher enculturation score was associated with higher stress (*r* = 0.20, *p*<0.001) and depression (*r* = 0.16, *p* = 0.004). These results demonstrate good convergent validity.

**Table 4:** Correlations of acculturation, enculturation, and traditional indicators for acculturation.

	1	2
1.Acculturation		
2. Enculturation	-0.01	
3. Age at immigration	-0.14*	0.15**
4. Duration of living in Taiwan	0.24**	-0.11*
5. Chinese language ability	0.40**	0.14*
6.Stress	-0.25**	0.20**
7.depression	-0.23**	0.16**

*Note*.

*p<0.05

**p<0.01.

## Discussion

The present study developed a bidimensional acculturation scale and assessed its psychometric properties. It includes both the dimensions of acculturation, adaptation to host culture (acculturation) and maintenance of heritage culture (enculturation), and focuses on Asian immigrant women in Taiwan. The findings showed that the 19-item BAS-MBIW with paralleled acculturation and enculturation subscales is a valid, reliable instrument to detect attitudes and behaviors toward host and heritage cultures in terms of the necessities of everyday life among pregnant immigrant women in Taiwan. We believe that the BAS-MBIW can be applied to marriage-based immigrant women who migrate to Asian patrilineal countries though further validation studies may be needed.

EFA and CFA supported the construct validity of the bidimensional acculturation scale. The acculturation and enculturation subscales include six latent factors: language use, media use, food preference and use, cultural heritage, social interaction, and shopping and merchandise preference. This multi-faceted structure is consistent with previous bidimensional acculturation scales developed for use in the United States [19, 28, 29, 31, 32]. Based on our findings, the correlation coefficients between the six latent factors were, on average, low in both subscales, which suggests that each latent factor is unique and important. For example, language use in communication, thinking, and message reception reflects an instinct or a skill. However, language use did not completely correspond to attitudes and behaviors related to eating, shopping, cultural heritage, and social interaction in the host country, even though daily life behaviors showed a strong connection with language [58, 59]. Both acculturation and enculturation subscales fitted the second-order six-factor models in CFA. All six domains loaded well on acculturation and enculturation subscales (factor loading >0.3); except for the path between language use and enculturation (factor loading = 0.11). This finding also suggests that mother language use may not be strongly related to maintenance of heritage culture, though the association is statistically significant. This may be due to those marriage-based immigrants who have a good command of the local language or who experience a lack of resources to maintain their heritage culture in Taiwan.

Several scales have been designed to measure the bi-dimensional acculturation of Chinese American (General Ethnicity Questionnaire-Abridged), (East) Asian American (East Asian Acculturation Measure [EAAM] and Stephenson Multigroup Acculturation Scale), or both (Vancouver Index of Acculturation) [19, 28, 31, 32]. A previous study translated the EAAM into Chinese (EAAM-C) and performed psychometric testing among immigrant women in Taiwan [33]. The EAAM-C retained 15 out of the 29 original EAAM items. The EAAM items focused on language/media use and social interaction domains and the researchers were more interested in patterns rather than the level of bi-dimensional acculturation [28, 33]. The aforementioned scales do not include cultural and traditional living items that are deemed important to Taiwanese culture. Since marriage-based immigrant women in Taiwan usually assume full responsibility for household work and family care, they are customarily involved in festival cooking, shopping and meal preparation in the mainstream society. Therefore, besides language/media use and social interaction, our scale also incorporates domains of food culture, cultural heritage (custom, folk belief, and festivals), and shopping and merchandise preference to comprehensively capture their acculturation level.

In the uni-dimensional version of the concept, acculturation is usually assessed by proxy variables, including language ability and exposure to the host culture (e.g., duration of living in the host country and age at immigration). Better language ability and a higher level of exposure to a host culture generally represent a higher level of adaptation to a host culture [36, 60, 61]. This study found that immigrant women who had better Chinese language ability, had lived in Taiwan longer, and were younger at immigration were more likely to adapt to their host culture. This study confirmed that these key immigration-related variables are associated with an individual’s level of acculturation, which is consistent with the findings of previous studies [23–25]. This similarity indicates an acceptable convergent validity for the acculturation subscale.

Few studies have evaluated the association between an enculturation scale and the above-mentioned proxy indicators for acculturation. Since married women are generally expected to accept the customs of their husbands’ families in Taiwan, we expected immigrant women who had lived in Taiwan longer to encounter difficulty in maintaining their heritage culture. In addition, being older at immigration means an individual has had a longer duration of exposure to her heritage culture. Therefore, we hypothesized that immigrant women who had lived in Taiwan longer would be less likely to maintain their heritage culture, while immigrant women who were older at immigration would be more likely to maintain their heritage culture. Our findings supported both speculations, indicating an acceptable convergent validity for the enculturation subscale. However, this study found that immigrant women who had better Chinese language ability were more likely to adapt to the host culture and maintain their heritage culture. This finding may because that majority of participants were from China (68% in the study). The language and culture of China is more similar to Taiwan than those of other countries.

Higher acculturation and lower enculturation were associated with lower stress and depression among pregnant immigrant women in this study. This finding is in line with theories that high acculturation may enforce social integration [62] and awareness of health-related options [63, 64]. A previous study also indicated that a lower level of adaptation to host culture was associated with higher prenatal depression [57]. However, our finding of the correlation between enculturation and prenatal depression is inconsistent with previous studies that indicate no statistically significant correlation [57, 65]. Immigrant women who endeavor to maintain their heritage culture in Taiwan may experience conflicts between heritage and mainstream prenatal cultures and face challenges due to the absence of a heritage culture-friendly environment, which may lead them to feel stressed and depressed. Further studies may be needed to test this proposition.

Based on our findings, adaptation to host culture and maintenance of heritage culture are not correlated, which supports the theory that acculturation has two independent dimensions. Immigrant women in Taiwan often face discrimination and misunderstanding from their husbands’ families and Taiwanese society [66]. Therefore, it is necessary to advocate the bidimensional concept of acculturation to revive immigrants’ cultural background as their strength and promote cultural pluralism. Measurements from the bi-dimensional acculturation scale can enable researchers, health professionals, and policymakers to better understand cultural pluralism, provide resources and strategies more efficiently, and improve the well-being of immigrant populations. The finding that bi-dimensional acculturation level is related to stress and depression suggests that healthcare workers could use the scale to assess their immigrant patients and provide assistance accordingly in order to decrease stress and depression among immigrant women. Future studies could use the BAS-MBIW and examine the effect of bi-dimensional acculturation on acculturative stress, health outcomes, and healthcare utilizations. The BAS-MBIW could be applied regularly in order to assess the efficacy of policies of acculturation for immigrants.

This study has several limitations. The study participants were a convenience, homogeneous sample of marriage-based pregnant immigrant women in Taiwan. Only five of the participating women were from Western or Northeast Asian countries. Nonetheless, the percentages of the birth countries in this study (China: 68%; Vietnam: 16%; other Asian countries: 15%) were similar to national data in Taiwan (China: 67%; Vietnam: 21%; other Asian countries [Indonesia, Philippines, Thailand, Cambodia, Japan, and Korea]: 11%) [4]. Future studies could address this concern by evaluating marriage-based immigrant women in other Asian countries or re-examining the psychometric properties in Taiwan. The data relied on self-report through interview to detect and decrease potential language barriers. Immigrant women may thus have responded in socially desirable ways due to a lack of anonymity. A longitudinal design or measurement at different life stages is needed to further validate the scale and explore the relationship between bidimensional acculturation and health outcomes.

## Conclusions

This valid, reliable bidimensional acculturation scale is the first instrument developed for East Asian immigrant women in a modern Chinese society. The BAS-MBIW includes multiple domains (language use, media use, food preference and use, cultural heritage, social interaction, and shopping and merchandise preference). It provides comprehensive measurement and detects attitudes and behaviors toward host and heritage cultures in terms of the necessities of everyday life. The BAS-MBIW could be used to assess bi-dimensional acculturation among marriage-based immigrant women.

## Supporting information

S1 Data (SAV)
